# The Development and Implementation of a Veteran Family Stress Screening Tool

**DOI:** 10.3390/healthcare14101335

**Published:** 2026-05-13

**Authors:** Valentina Stoycheva, Katelyn C. Vala, Rebecca M. Schwartz, Juliet M. Vogel, Peter J. D’Amico, Mayer H. Bellehsen

**Affiliations:** 1 Stress and Trauma Evaluation and Psychology Services, New York, NY 10168, USA; valentina.stoycheva@gmail.com; 2 Northwell Health, New Hyde Park, NY 11040, USA; rschwartz3@northwell.edu (R.M.S.); jm_vogel@verizon.net (J.M.V.); pdamico@northwell.edu (P.J.D.); mbellehsen@northwell.edu (M.H.B.); 3 Department of Psychiatry, Donald and Barbara Zucker School of Medicine at Hofstra/Northwell, Hempstead, NY 11549, USA; 4 Department of Occupational Medicine, Epidemiology and Prevention, Donald and Barbara Zucker School of Medicine at Hofstra/Northwell, Hempstead, NY 11549, USA

**Keywords:** Veteran mental health, Veteran families, military families, military children, referrals, health service delivery, PTSD

## Abstract

**Highlights:**

**What are the main findings?**

- A structured screening tool implemented within the Veterans Health Administration captured Veterans’ self-reported perceptions of relationship difficulties and child developmental, behavioral, and emotional concerns within their families.
- Over one-third of screened Veterans requested follow-up support, and many were connected to referrals, indicating perceived family needs that may be addressed through available resources.

**What are the implications of the main findings?**

- Integration of family-focused screening within Veteran healthcare settings is feasible and acceptable.
- Routine family-focused screening within Veteran healthcare settings can improve early identification of psychosocial and child-related concerns that are often overlooked.
- 41.2% of screened Veterans requested follow-up support, and 57.4% of those received referrals.

**Abstract:**

**Background/Objectives**: Military and Veteran families face unique challenges, including deployment-related difficulties and transitions, mental health issues, educational setbacks, and more. The needs of Veteran families, specifically, are often overlooked and research among this population is insufficient. In an effort to promote the well-being of children of Veterans and their families, we developed a screening tool for identifying family needs and a workflow to implement the screening tool within the Veterans Health Administration. The objective of this quality improvement initiative was to then provide those in need with appropriate referrals and connect them to timely care. **Methods**: The screening tool was developed with input from key stakeholders and adjusted after an initial pilot. Screens were offered to all Veterans seen at the participating sites. Veterans completed the self-report screen consisting of demographic information and items regarding household composition, the quality of relationships with family members, and the Veteran’s perceptions of difficulties experienced by family members. Descriptive statistics were conducted to summarize the data with regard to demographics, relationship difficulties, and related needs of Veteran family members. **Results**: Among familial relationships, Veterans reported experiencing the most difficulties with their partner/spouse as compared to other family members. Veterans self-reported that many of their children 18 years old and younger demonstrated difficulties related to learning, development, and behavioral and emotional concerns. **Conclusions**: More than a third of Veterans screened in this process requested further contact for consultation and referrals, and half of those were provided referrals. The development and implementation of this screening tool and referral procedure was successful in identifying needs and facilitating a connection to care that might otherwise have not occurred, bridging the gap between research and practice as it relates to Veteran family needs.

## 1. Introduction

According to the U.S. Department of Veterans Affairs Veteran Population Projection Model (2020), as of 2020, there were over 19.4 million military Veterans in the population [1]. Per a report from 2016, the majority of Veterans were married (63.3%), constituting about 11.5 million Veteran families [2]. Altogether, 13.4% of Veterans lived with children in their home. This statistic substantially increases when looking at the post-9/11 Veteran cohort specifically, in which approximately 43.8% live with children in their home [2]. While the target population of the current study is that of Veterans who have since separated from the military, there is very limited research on children and families of this specific subpopulation. Thus, research pertaining to active service members and their families will be reviewed as these challenges remain relevant and can be extrapolated to Veteran families, as all Veterans were at some point on active duty. Statistics from the Department of Defense indicate that there are nearly 2 million children of a service member [3]. While military families exhibit resilience, they are known to face unique challenges, especially when compared to non-military civilian families [4].

The primary challenge in studying the children of Veterans is the lack of a systematic method to identify and track this population. In addition, there is currently no systematic, standardized process for the screening of family stress within the VA system. Although some studies have utilized the National Survey of Children’s Health, a nationally representative database that now includes questions about caregiver military or Veteran status, it is uncommon to find a national database that incorporates information specific to this population. Even when such data is available, it typically does not allow for the identification of individual children, which hampers efforts to address their specific needs and provide necessary services. This lack of identification also limits the ability of healthcare systems, including the Veterans Health Administration, to systematically screen for family stressors or connect Veteran families with appropriate support services. Regardless, all children of parents who have deployed in the past are subject to the impact of parental mental health and readjustment issues, and the discussion of post-deployment military families typically includes the time when the service member transitions to Veteran status.

Military service is often accompanied by significant stressors for both Veterans and their families, including deployments, frequent relocations, and uncertainty regarding the service member’s safety [5,6]. Family relocations require adjustment to new homes, schools, and communities [5], and military children change schools an average of six to nine times between kindergarten and high school graduation [7]. These transitions may affect psychosocial adjustment and the ability to establish and maintain peer relationships [8].

While many Veterans may start families post-service, many others transition from military life into Veteran life as a family unit. When they cross over to Veteran status, the family challenges created by service remain. The Deployment Life Study, a three-year longitudinal study examining the cycle of deployment, assessed outcomes related to quality of relationships, well-being, military integration, and the psychological, behavioral, and physical health of family members [9]. While service members reported improved conditions in their family environment and higher parenting satisfaction during deployment, their spouses reported no change in family environment and declines in parenting satisfaction [9]. Over the course of deployment, marital satisfaction decreased, as did the quality of relationships between adolescents and the deployed family member. As of 2010, one in four military children experienced depression and one in five experienced academic problems [10]. Although evidence suggests that military children demonstrate resilience despite these challenges, studies indicate that resiliency increases when adequate support systems are in place [10], highlighting the importance of support services and resources for military children and families.

Deployment status has been shown to have a potentially negative impact on both the service member and their family. Veterans who have deployed are at an increased risk of PTSD, which has been linked to increased individual distress for the Veteran, as well as disruptions in family functioning [11]. For example, in comparison to those without PTSD, male Veterans with PTSD have demonstrated markedly elevated levels of family violence, increased difficulties with intimacy, and greater relationship distress [12,13]. Common symptoms of PTSD that negatively impact familial relationships include emotional numbing, anger, and avoidance, which contribute to a lack of engagement and aggression within the home [14,15]. The shift from active duty to civilian status can also be especially difficult for some Veterans. Loss of military identity, reintegrating into communities, and creating a purposeful life contribute to strain associated with the transition. These individuals are at an increased risk for depression, risky behavior, and homelessness [16].

In a recent qualitative analysis, spouses of Veterans diagnosed with PTSD provided their perspectives, highlighting the challenges they face that contribute to dissatisfaction within the family. They shared experiences of emotional detachment and rejection and expressed an overall need for both social support and caregiver support [17]. These findings provide evidence regarding the stressors endured by spouses of Veterans and highlight the need for family support. 

In addition to spouses, children and adolescents between the ages of 10 to 15 of Veteran fathers with PTSD were found to exhibit the following concerns: emotional difficulties, behavioral problems, hyperactivity and impulsivity, issues with their peers, and an overall elevated subjective stress level [18]. Additionally, children whose Veteran parents have PTSD report twice as many internalizing and externalizing problems, as compared to children of Veteran parents without PTSD [19]. These issues include anxiety, depression, somatic complaints, rule breaking behavior, aggressive behavior, attention problems, and more [19]. Likewise, children of Veteran families specifically have been found to demonstrate substantially higher rates of externalizing problems (i.e., behavioral or conduct issues) in comparison to children of nonveterans, and are more likely to be diagnosed with ADD/ADHD or a substance use disorder [20].

Due to the many transitions and challenges faced by Veterans and their families, the mental health needs of the entire family unit are especially important. The U.S. Department of Veterans Affairs offers support for family members of eligible Veterans through several programs designed to provide mental health services, caregiver support, and access to resources [21]. Despite expansions and improvements to these programs, there remains a continued demand for greater utilization of supports and additional resources, as parents of military children continue to report developmental and family-related challenges [8,10,22,23]. It is clear that further support for Veteran families and children is warranted, as the impact of deployment and other service-related challenges extend beyond physical transitions to impaired social, emotional, behavioral, and academic functioning. An important first step in increasing connections between Veteran family members and much needed resources is to effectively identify those who could benefit from services and the type of services they may seek [24]. Despite these challenges, there are currently minimal systematic efforts within the Veterans Health Administration to screen for or identify the needs of Veteran family members.

This is the case, even though research indicates that mental health screenings for Veterans themselves have been beneficial in connecting them to timely care. More specifically, studies have found that implementing screening for deployment-related conditions was strongly associated with shortened delays to receiving care compared to those who were not screened, with the median delay being shorter by about one year [25]. To address these gaps, the items included in our screening tool were directly mapped to key stressors identified in the literature, including partner distress, child behavioral and academic difficulties, and service-related challenges. Unlike existing Veteran only screens, this tool adopts a family systems lens, capturing both the Veteran’s experiences and their perceptions of family members’ needs. By doing so, the screen is designed to identify actionable family needs and support timely connection to relevant resources.

The present quality improvement (QI) project intended to extend this screening process to Veteran family needs as well. In an effort to promote the well-being of children of Veterans and their families, we aimed to develop a screening tool for identifying family needs and workflow to implement within the Veterans Health Administration. Based on the gaps in the literature for this group, we included items related to family composition, potential developmental and emotional challenges that could be experienced by Veterans’ children, the stress experienced by the family members, and the stress experienced by the Veteran due to challenges with family members. The initial item development was informed by Family Systems Theory so that it was inclusive of the interconnection and interdependence of behaviors within the family system [26]. The screen and follow up process would allow for a more thorough understanding of the needs of Veteran families as perceived by the Veteran, and linkage to services. Specifically, we aimed to screen Veterans regarding their own experiences within their family relationships as well as their perception of their loved ones’ experiences and to then provide appropriate referrals based on their responses. Upon identification of these families, we provided Veterans who expressed interest with referrals that ranged from behavioral health services to academic supports. The current study is a descriptive evaluation of the screening process.

## 2. Materials and Methods

***Survey Development.*** Administrative and clinical staff from one of the Veterans Administration Medical Center (VAMC) Outpatient Clinics collaborated with staff at a community-based clinic for Veterans and their families to develop both the Military and Veteran Family Stress Screen and a process for its administration. This was determined to be a Quality Improvement project as per the Institutional Review Board and therefore given exemption status (#18-0653). The screen was a self-report screen utilized to identify the needs of Veterans and their families. The screen was administered between March 2018 and March 2020. Prior to approval, the screen was shared with the Veterans Mental Health Council (VMHC) [27]. The VMHC consists of mental health providers who work directly with Veterans and their family members, representatives of the VA, the community, and consumers of services. After the VMHC reviewed the screen and deemed it appropriate and feasible for use, it was internally vetted at the VAMC by a forms committee, assigned a form name, and finally approved to be utilized within that VA’s Outpatient Clinic with patients. The forms committee ensured that it was brief in terms of the number of items, that the language level was appropriate and would be readily understood, and that the items were not too sensitive in nature. A Without Compensation (WOC) psychologist employed by a large healthcare system was placed as a liaison at the VA one day a week to assist direct Veteran care providers in the rolling out of the screen, as well as to provide resources to Veterans for their identified needs. The screening and follow up process was as follows.

The survey was added to the intake packets for Veterans entering the U.S. Department of Housing and Urban Development-Veterans Affairs Supportive Housing (HUD-VASH) and Caregiver Support programs, as well as for Veterans seeking mental health services through the Women’s Health program. Existing clients were identified for screening at their next regularly scheduled appointments, with the goal of completing the screen once a year. 

***Procedure.*** At the time of visit, the Veteran was given a letter describing the purpose of the screen along with the screen to complete and return to the provider (e.g., psychologists, social workers, postdoctoral fellows). This was given to all Veterans who were seen by the psychologists, social workers, and fellows who were part of the screening program initiative, resulting in a convenience sampling structure. Veterans were not compensated for completion of the screen. After review, if items of concern were present, such as risk of harm or domestic violence, the clinicians were to follow VA protocols for next steps. If items of concern were not present, the clinician utilized the information obtained from the screen as part of care formulation for the Veteran. The screen was scanned into the client’s chart, along with a general statement regarding the screen outcome, for example, “Veteran completed Military and Veteran Family Stress Screen and endorsed some difficulties dealing with daughter’s behavior. The [WOC] psychologist is being alerted to follow up with Veteran.” Then, if a follow up was warranted, the WOC psychologist either made a phone call to the client to provide further information that was requested or followed up in person, providing referrals for resources as needed and available. WOC psychologists made three attempts at reaching the Veterans who self-identified as needing resources. Referrals were made that were specific to the challenges identified by the screen (e.g., marital counseling, educational resources for children).

The second stage of rollout of the Veteran Family Stress Screen included a similar workflow but expanded the utilization of the screen within the OEF/OIF/OND Clinic, as well as the VA Community-Based Outpatient Clinic (CBOC) and a community-based clinic that was partnered with the VA. In addition, the screen itself was revised for clarity based on input from the psychologists who were implementing it (see Appendix A). During the second stage of the administration of the screen, community staff worked with VA staff to advocate for a wider utilization of the screen across services, as well as to create an electronic version of the screen to be incorporated into the Veterans’ electronic medical records to facilitate ease of integration into the EMR.

***Measures.*** The initial approved screen was comprised of questions regarding the Veteran’s family and household composition, experience of difficulties within each of these relationships, and the Veteran’s perception of their family members’ difficulties. The latter were reported on a Likert scale of 0–3 where 0 indicated no difficulty and 3 indicated very high difficulty. Specific challenges experienced by Veterans’ children (e.g., behavioral, emotional, learning, or developmental, etc.) were also assessed. The screen was later revised after administration to 76 participants to include questions related to age, deployment status, and the year joined and year separated from military. Additional questions were added to specify whether or not the challenges experienced were perceived to be military service related. In addition to the demographic information collected, the final screen consisted of eight items. The total number of Veterans approached to be screened in both stages of the screen administration was 217.

***Data Safety.*** Data were collected and stored on the lead psychologist’s secure, password protected computer within the hospital. Once de-identified by the psychologist, the data was shared on a password protected shared folder with the data analyst. Data is retained by the psychologist as it is part of the clinical record; however, data will be destroyed by the analyst after 10 years.

***Analytic Approach.*** Descriptive statistics were performed to describe the sample demographics as well as relevant items on the screen, such as levels of difficulty reported in relationships and challenges faced by children of military families. Means and standard deviations were used to describe continuous variables while frequency and percentages were used to describe categorical variables. All data analysis was conducted through IBM SPSS v27.

## 3. Results

A total of 217 Veterans were approached, and 174 participated in the screen (80% response rate). Quality assurance facilitated no missing data. Of those who reported age, the mean was 43.31 (*SD* = 14.84) indicating that the sample spanned various deployment cohorts. The majority were male (69.8%). Regarding racial and ethnic background, 41.4% of participants were non-Hispanic White, 23.5% African American, 21% Hispanic/Latino, and the remaining participants were Asian, Native Pacific Islander, American Indian/Alaska native, or two or more races. Of those who reported deployment status, 56.7% were deployed with combat exposure, 23.3% deployed with no combat exposure, and 20.0% were never deployed. Veterans were referred from one of the following sources: Caregiver Support (5.3%), HUD-VASH (41.2%), Women’s Health (14.7%), Operation Enduring Freedom/Operation Iraqi Freedom/Operation New Dawn community site (30.6%), and VA CBOC (8.2%). Demographics are presented in Table 1. 

A Veteran’s family composition included any of the following: a spouse or significant other, children, their family of origin, including parents and siblings, and grandchildren. Seventy-nine Veterans indicated that they have children 18 years and under in their families (45.4%). The number of children reported to be 18 years or younger totaled 139, 74.8% of whom live in the home of the Veteran and 25.2% of whom were not currently living with the Veteran. 

**Figure 1:**
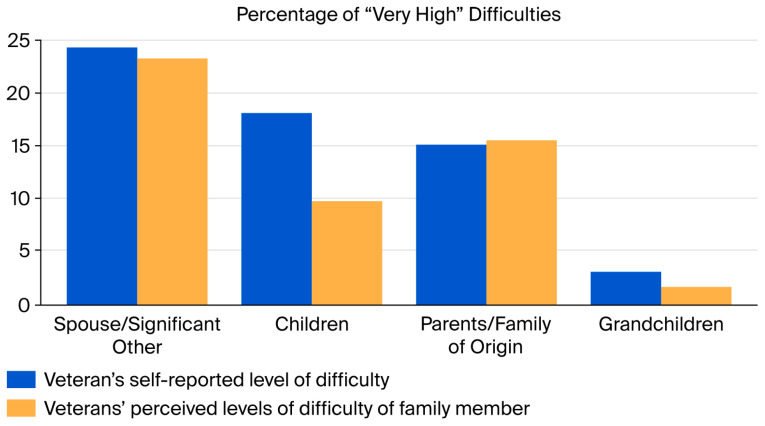
Percentage of “Very High” Difficulties.

Among all relationships, the Veterans reported having the highest level of difficulty overall with their spouse/significant other. Of Veterans who reported having a spouse/significant other (n = 139), 24.5% (n = 34) reported “Very High” levels of difficulties in these relationships. Similarly, of those that reported having a spouse and responded to questions about their stress (n = 128), 23.4% (n = 30) also reported their perceived spouse/significant other’s difficulties to be “Very High”. Of those Veterans with children (n = 115), 18.26% (n = 21) reported “Very High” levels of difficulties, while only 9.81% (n = 11) rated their children to have “Very High” levels of difficulty. These data, along with the percentage of those who reported “Very High” levels of difficulty among the remaining family members, are shown in Figure 1.

**Figure 2:**
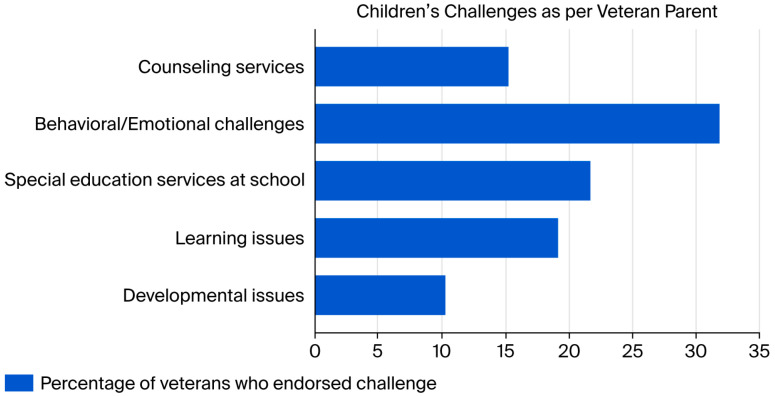
Children’s Challenges as per Veteran Parent.

Veterans with children under 18 years old (n = 79) reported a variety of difficulties they believe their child may be experiencing (Figure 2). Of the respondents who reported having children 18 and under, 31.65% (n = 25) reported that they had children with behavioral or emotional challenges, 18.99% (n = 15) reported that they had children with learning issues, and 10.13% (n = 8) reported that they had children with developmental issues. Respondents were able to indicate as many difficulties as applied to them. Regarding additional support, 21.52% (n = 17) of participants indicated their child was receiving special education services at school and 15.19% (n = 12) reported that their children were participating in counseling services. Moreover, of the 157 Veterans who answered the survey item, 27.4% (n = 43) indicated that they had children residing outside of the home with whom they would like to have a closer relationship, an indicator of relational strain. 

Of the more than half of Veterans who responded to the question regarding service-related difficulties (n = 67), 59.7% (n = 40) indicated that their difficulties are a result of service-related challenges such as deployment stress, reintegration stressors, financial difficulties, mental or medical health issues, and employment difficulties. Regarding the perceived difficulties of their family members, 40.3% (n = 27) of Veterans reported that they believed those challenges were a result of their service. Moreover, 29.6% (n = 24) of the 81 Veterans who responded to the item reported that they were concerned about the way in which their family members were coping with any service-related challenges. 

Of the 174 participants, 68 Veterans (41.2%) requested to be contacted for further consultation and resources. Of those 68 Veterans, 39 were provided referrals (57.4%) and 23 (33.8%) could not be reached. The remaining six (8.8%) indicated that a referral was no longer needed. 

## 4. Discussion

To our knowledge, this is the first effort to date aimed at deploying a screen for family stressors in the Veteran population to identify needs and provide referrals. This quality improvement initiative represents a descriptive evaluation of the implementation of the screening tool and referral workflow within the Veterans Health Administration and is an initial step in a larger effort to standardize and then more widely implement family stress screening in this setting. Veterans who completed our screen endorsed substantial levels of family stress related to military service. These Veterans self-reported that many of their children 18 years old and younger demonstrated academic and emotional difficulties and that they were experiencing high levels of stress regarding their family members. Further, 27.4% indicated a desire for closer ties with non-resident children, highlighting relational strain. More than a third of Veterans screened in this process requested further contact for consultation and referrals and half of those were provided referrals. These outcomes suggest that the development and implementation of our screening tool and referral procedure is feasible and promising in identifying perceived family needs and generating interest in further consultation or referrals. However, screening alone may not consistently result in completed connections to services and highlights the need for additional systems to support follow-up and care coordination.

There are several implications of our findings. Given the increased distress experienced by Veterans and their family members as it relates to interpersonal, emotional, behavioral, and academic functioning, the identification of family struggles and connection to resources is especially important. This project is an initial step in providing evidence of this need in this specific population, as opposed to the larger body of literature that focuses on active military families [8,9,10]. Thus, an effort to implement similar screens to detect Veteran family stress in mental health settings of the Veterans Health Administration system, such as the screen created in this project, may increase the number of Veterans that could be supported around their family needs. At the same time, the descriptive nature of this implementation project and the reliance on self-reported perceptions underscore the need for cautious interpretation of the findings. Given that bolstering support for the family can further foster resilience, behavioral health initiatives are necessary to address familial stressors [10]. Programs aimed at the behavioral health needs of Veteran children specifically are crucial, but care should be taken to also address the academic needs of this population. Additionally, the high rates of difficulties reported in partnerships demonstrate the need for initiatives aimed at couples in particular. Effective strategies could include treatment interventions for relationship problems, such as couples counseling, as well as training that targets skills for communication or provides psychoeducation on caregiver burden and post-traumatic stress. Integration into electronic health records (EHRs) and standardized referral workflows could improve follow-up and coordination of care, helping ensure identified needs translate into actionable support.

Several limitations should be noted. First, as this was a QI project, not a research study, and it did not utilize representative sampling procedures, our results may not generalize across other Veteran populations. For example, participants were recruited from specific programs (e.g., HUD-VASH and Women’s Health), which may not reflect the broader Veteran population. Furthermore, due to the data collection occurring a number of years ago, it is possible that referral pathways and needs may have changed over time and therefore may not be as generalizable today. Second, it is important to note that the data in the screen represent Veterans’ perceptions of difficulties, which may not accurately represent the reality of family members’ experiences. These perceptions reflect the Veteran’s perspective of family functioning. It is possible that direct screening of family members could have identified even greater (or different) concerns; however, it is also meaningful to utilize tools that capture the Veteran’s perception since they are often the person in treatment at a VAMC and their perception of family functioning may also impact their wellbeing. Moreover, as screening participation was voluntary, the sample may reflect Veterans who were more willing to engage in screening, which could introduce selection bias. Further, while there is preliminary indication of face validity to the items selected, the absence of validated scales and psychometric testing represents a central limitation of this screening approach and limits conclusions that can be drawn about measurement reliability or validity. Future research should include psychometric testing and refinement of the screen to establish reliability and validity. This would also enable hypothesis testing and inferential statistical analyses. Third, the screen underwent revisions after the initial administration to numerous participants, and thus data regarding age, deployment status, and other factors was not consistently collected throughout the sample. However, this reflects the real-world experience of developing and refining a tool in the clinical space. Lastly, our process only extended to the provision of referrals. We were not able to assess whether Veterans and their families ultimately connected to the services provided or their satisfaction with those services. Future work would benefit from incorporating follow-up procedures or referral tracking systems to determine whether identified needs result in successful service engagement.

Given the various challenges experienced by Veterans and their families, this effort demonstrates the value of utilizing a screening tool to not only capture the extent of the distress within familial relationships and identified needs, but also to serve as a catalyst for change by creating linkages to supports. Additional work is needed to strengthen referral pathways and follow-up processes so that identified needs can more consistently translate into meaningful support for Veterans and their families. It remains clear that family support services are needed for this population and that increased integration of a screen and appropriate follow up into Veteran facing systems like the Veterans Health Administration could facilitate an increase in support for these families.

## 5. Conclusions

The present study sought to better understand and respond to the family needs of Veterans by implementing a structured screening process within the Veterans Health Administration. Findings indicate that many Veterans experience notable stress within family relationships and report concerns related to the developmental, behavioral, and emotional functioning of their children. The screening process was preliminarily feasible and acceptable for use, although without formal survey validation, additional feasibility testing will be required. It provided an initial step toward the development of a practical method for identifying these concerns and offering referrals to Veterans who expressed interest in additional support. While further work is needed to strengthen referral pathways and evaluate longer-term outcomes, these findings are a first step toward incorporating family-focused screening into Veteran healthcare settings in order to bring greater attention to the needs of Veteran families and inform efforts to connect them with appropriate resources.

## Figures and Tables

**Table 1:** Sample Characteristics *.

Variable	Total *N* = 174
Age in Years, Mean (range; SD)	43.31 (20–84; 14.8)
Gender	
Male	118 (69.8)
Female	51 (30.2)
Race/Ethnicity	
African American	38 (23.5)
American Indian/Alaska Native	1 (0.6)
Asian	4 (0.6)
Hispanic/Latino	34 (21.0)
Non-Hispanic White	67 (41.4)
Native Hawaiian/Pacific Islander	2 (1.2)
Two or more races	16 (9.9)
Deployment Status	
Never deployed	12 (20.0)
Deployed, no combat	14 (23.3)
Deployed, combat	34 (56.7)
Children	
N (%) of Veterans who have a child < 18	79 (45.5)
Number of children < 18 (% out of total number of children)	139 (32.9)
Number of children > 19 (% out of total number of children)	283 (67.1)
Number of children < 18 living in the home (% of 139)	104 (74.8)
Number of children < 18 not in the home (% of 139)	11 (7.9)
Number of children < 18 part time in the home (% of 139)	24 (17.3)

* Numbers may not add up to the total N due to missing data.

## Data Availability

The datasets produced for this current study are available upon reasonable request.

## Abbreviations

The following abbreviations are used in this manuscript:

VA	Veterans Administration
PTSD	Post-Traumatic Stress Disorder
PCAFC	Program of Comprehensive Assistance for Family Caregivers
PGCSS	Program of General Caregiver Support Services
VAMC	Veterans Administration Medical Center
VMHC	Veterans Mental Health Council
WOC	Without Compensation
HUD-VASH	Housing and Urban Development-Veterans Affairs Supportive Housing
CBOC	VA Community-Based Outpatient Clinic
OEF/OIF/OND	Operation Enduring Freedom/Operation Iraqi Freedom/Operation New Dawn

## Appendix A

**Please fill out the following sections accurately, and to the best of your knowledge and ability. Keep this document safe in your possession, and give the completed version to the clinician at the time of your interview. Please be advised that this document will be reviewed with you during your appointment. The clinician will document the review in your medical record, and this document will be scanned into your medical record in its entirety.**

Name: __________  Last Four of Social Security: ________  Date: ______

**Military & Veteran Family Screen**

**Gender:**	___Male		___Female	___Transgender	___Other
**Age:** ________	**Year joined military:**________			**Year separated from military:**___________	
**Race/Ethnicity** (Circle One)	African American American Indian/Alaska Native		Asian Hispanic or Latino	Non-Hispanic White Native Hawaiian/Pacific Islander	Two or more races
**Deployment status** (circle one): Never Deployed Deployed; no Combat Exposure Deployed; Combat Exposure					

**1. Who is in Your Immediate Family?**			**Lives at Home?**		
First Name	Age	Relationship	Yes	No	Only part-time
					
					
					
					
					
					

**2.** Do you have children not living in your home with whom you would like to have a closer relationship?

  **Yes   No**

**3.** Do you have any children between the ages of 0–18 with any of the following (please circle all that apply)

(a) Behavioral or emotional challenges
(b) Learning issues
(c) Developmental issues
(d) Receiving special education services at school (e.g. Individualized Education Plan (IEP), 504 accommodations, or special school placement).
(e) Receiving early intervention (EI) services
(f) Counseling services

**4.** Using the grid below, please rate the level of difficulty you are experiencing in your relationships with the following individuals (e.g., problems with school or work, conflict, communication difficulties, fears/anxiety, sadness, anger, other behavioral or emotional issues, difficulty with caregiving for children/loved ones):

Relationship	Level of Difficulty				
	0 None	1 A little	2 Moderate	3 Very High	Not Applicable
Spouse/significant other					
Child #1					
Child #2					
Child #3					
Parents/Family of origin					
Grandchildren					

**5. Are *any*** of the above difficulties ***you* are experiencing** related to or a result of service-related challenges like deployment stress, reintegration stressors, financial difficulties, mental or medical health issues, employment difficulties?

  **Yes   No**

**6.** Using the grid below, please rate the level of difficulties that ***your loved ones* may be experiencing** (e.g., problems with school or work, conflict, communication difficulties, fears/anxiety, sadness, anger, other behavioral or emotional issues, difficulty with caregiving for children/loved ones):

Relationship	Level of Difficulty				
	0 None	1 A little	2 Moderate	3 Very High	Not Applicable
Spouse/significant other					
Child #1					
Child #2					
Child #3					
Child #4					
Child #5					
Parents/Family of origin					
Grandchildren					

**7.** Are ***any*** of the above difficulties ***your loved ones* may be experiencing** related to or a result of service-related challenges like deployment stress, reintegration stressors, financial difficulties, mental or medical health issues, employment difficulties?

  **Yes   No**

**8.** Would you like someone to contact you or your family member for a consultation on ways that you or your family members can be helped?

  **Yes   No**

**Person/Phone # to contact: _________________________**

**Note:** Please be aware that in order to best assist you, many of the additional services we suggest may be in the community and, as such, are not VA services.
